# Exploiting the potential of extracellular vesicles as delivery vehicles for the treatment of melanoma

**DOI:** 10.3389/fbioe.2022.1054324

**Published:** 2022-11-17

**Authors:** Chongchao Hou, Qiang Wu, Lizhou Xu, Rongwei Cui, Rongying Ou, Danyang Li, Yunsheng Xu

**Affiliations:** ^1^ The Seventh Affiliated Hospital, Sun Yat-sen University, Shenzhen, China; ^2^ ZJU-Hangzhou Global Scientific and Technological Innovation Center, Hangzhou, China; ^3^ Department of Gynaecology and Obstetrics, The First Affiliated Hospital of Wenzhou Medical University, Wenzhou, China

**Keywords:** extracellular vesicle, drug delivery, melanoma, endogenous miRNAs, chemotherapy, immunotherapy, photothermal therapy (PPT), radiotherapy

## Abstract

Melanoma, the most aggressive skin cancer that originated from genetic mutations in the melanocytes, is still a troublesome medical problem under the current therapeutic approaches, which include surgical resection, chemotherapy, photodynamic therapy, immunotherapy, biochemotherapy and targeted therapy. Nanotechnology has significantly contributed to the development of cancer treatment in the past few years, among which extracellular vesicles (EVs) are nanosized lipid bilayer vesicles secreted from almost all cells that play essential roles in many physiological and pathological processes. In terms of melanoma therapy, the unique physicochemical properties of EVs make them promising nanocarriers for drug transportation compared to other synthetic nanocarriers. Moreover, EVs can be further engineered to maximize their drug delivery potential. Herein, in this minireview, we gave a brief overview of EV-based drug delivery strategies for melanoma therapy, in which different therapeutics delivered *via* EVs were summarized. We also highlighted the current progress of the EV-based delivery platform for melanoma therapy in clinical trials. The obstacles to applying exosomes in clinical practice toward further translation of EVs melanoma therapy were also discussed at the end. In summary, EVs offer promising prospects for melanoma therapy, whilst the ways for unlocking EVs’ full potential in melanoma therapies should be further investigated by solving relevant issues which hamper EVs-based melanoma therapy translation in the future.

## Introduction

**FIGURE 1:**
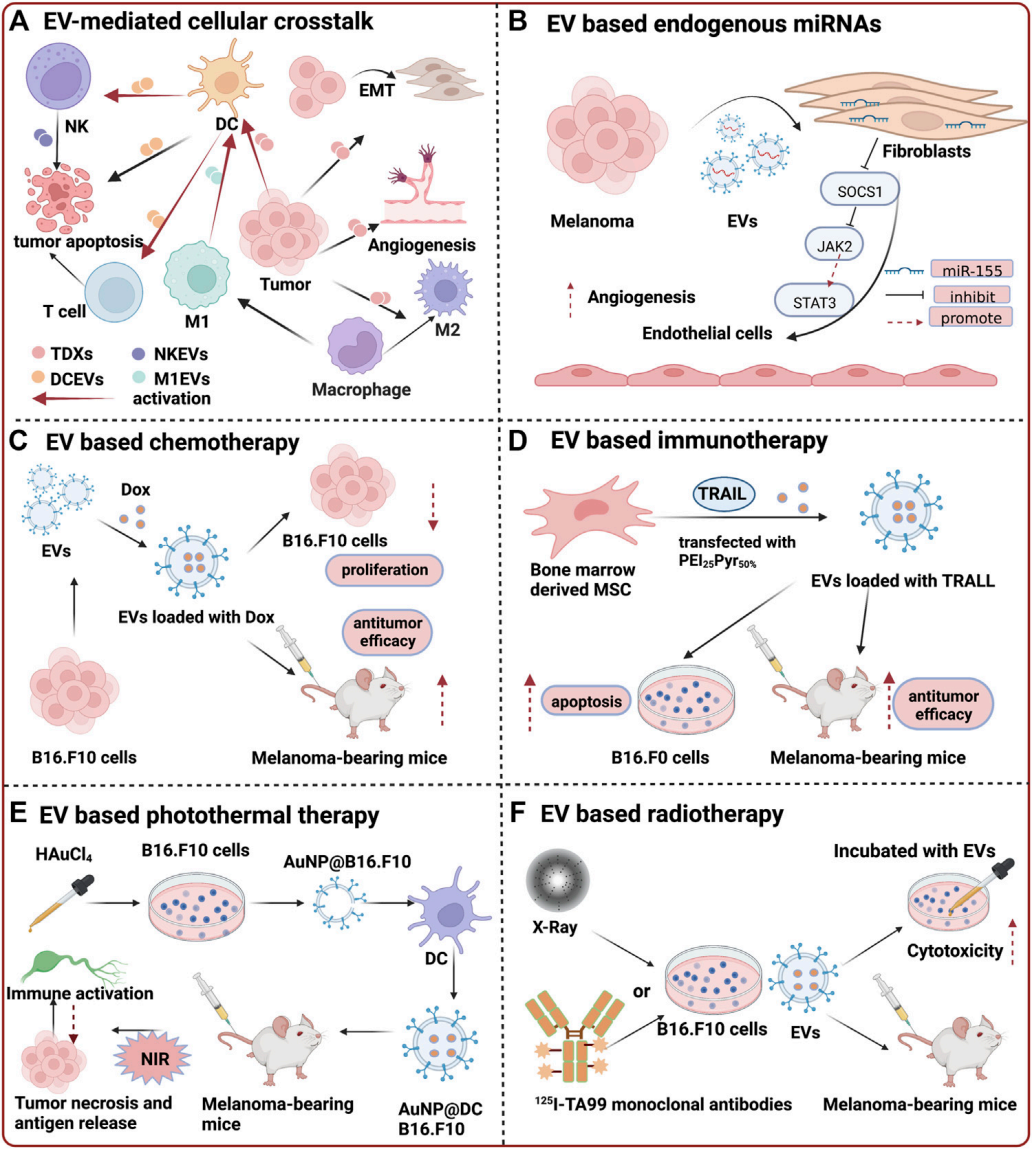
Summary of extracelluar vesicle assisted therapy for melanoma. **(A)** EV-mediated cellular crosstalk within the tumor microenvironment; **(B)** EV based endogenous miRNAs for melanoma treatment. MiR-155 within exosomes can induce the proangiogenic switch of cancer-associated fibroblasts by promoting the expression of proangiogenic factors *via* SOCS1/JAK2/STAT3 signaling pathway. (Zhou et al., 2018).Reproduced with permission. Copyright 2018, BioMed Central. **(C)** EV based chemotherapy. DOX that are loaded inside EVs exerted much higher inhibitory effects on the proliferation of B16.F10 melanoma cells. (Patras et al., 2022). Reproduced with permission. Copyright 2021, Taylor & Francis Group, LLC. **(D)** EV based immunotherapy. Bone marrow-derived MSCs transfected with plasmid encoding TRAIL indicated significant cell death compared to non-treated B16F0 cells. The tumor volume was also significantly reduced *in vivo*. (Shamili et al., 2018).Reproduced with permission. Copyright 2018, ELSEVIER. **(E)** EV based photothermal therapy. With near infrared (NIR) laser irradiation, AuNP@DCB16.F10 at tumor site generated hyperthermia, resulting in the apoptosis and necrosis of primary tumor and release of tumor antigens. Upon activation, mature DCs presented antigens to T cells and trigger the subsequent antitumor immune response. (Zhang et al., 2019a).Reproduced with permission. Copyright 2019, American Chemical Society. **(F)** EV based radiotherapy. EVs isolated from conditioned medium of Auger-RIT exposed cells are associated with about 30–40% bystander cytotoxic effects. (Karam et al., 2021).Reproduced with permission. Copyright 2021, Taylor & Francis Group, LLC.

Melanoma is a challenging skin cancer due to its high metastasis and chemoresistance. (Bandarchi et al., 2010). Current therapeutic systems for melanoma include surgical resection, chemotherapy, photodynamic therapy, immunotherapy, biochemotherapy, and targeted therapy. (Garbe et al., 2011). For melanoma at the early non-metastatic stage, surgical resection is preferred, and the prognosis is usually satisfying. However, the prognosis significantly worsens when regional lymph nodes are involved or metastasized. Although targeted therapy and immunotherapy have shown great promises in patients with advanced melanoma, drug resistance and related adverse effects to the above therapeutic measures could also not be neglected. (Chen et al., 2022). Thus, new methods to treat melanoma are urgently needed. In recent decades, nanomedicine has contributed remarkably for the development of cancer treatment. (van der Meel et al., 2019). Despite the fact that synthetic nanocarriers, including polymers, inorganic and biomimicking nanoparticles *et al.*, are increasingly widely used in cancer treatment, limitations such as their toxicity and low biocompatibility still hinder their further clinical applications. Extracellular vesicles (EVs) are lipid bilayer vesicles of nanometric size secreted most by all cells. Several subtypes of EVs, including exosomes, ectosomes, membrane vesicles, and apoptotic bodies, have been identified. (Witwer and Thery, 2019) (Herrmann et al., 2021). EV-mediated cellular crosstalk within the tumor microenvironment is complex, for example, some EVs from the same cell source may play dual roles, either inhibiting or promoting the growth of cancer. (Figure 1A). Specifically, EVs derived from tumor and immune cells (dendritic cells, macrophages, natural killer cells, etc*.*) are the most frequently studied. In terms of tumor-derived EVs (TDEVs), in one aspect, it can promote tumor growth, invasion and metastasis through multiple mechanisms, such as inducing epithelial to mesenchymal transition, creating a pre metabolic niche, promoting angiogenesis and macrophages polarization towards a tumor supporting phenotype, etc*.* (Sun et al., 2018; Ahmadi and Rezaie, 2020). Meanwhile, it can also transfer antigens from parental cells to indigenous immune cells, activate dendritic cells (DC) and further activate DC induced killer cells, resulting in anti-tumor effects. (Wang et al., 2020).Among immune cells, DC derived EVs (DCEVs) can activate natural killer (NK) cells and T cells to start the immune killing process as well as directly kill tumors, thereby playing an anti-tumor role. (Munich et al., 2012; Fu et al., 2022).Similarly, NK cell derived EVs (NKEVs) induce endogenous and exogenous apoptosis of cancer cells by presenting perforin and FasL. (Zhu et al., 2018). As for macrophages, M1 subtype derived EVs (M1EVs) can promote DC maturation, and further activate T-cell responses, activating anti-tumor response, while the M2 type exerted the opposite effects that inhibited the immune response. (Du et al., 2020). In combination of their multirole played in the tumor microenvironment and the cell-derived characteristics, EVs possess many advantages compared with synthetic nanocarriers, such as high biocompatibility, low immunogenicity, complex molecules transport ability, intrinsic active targeting and information transferring between different cells. (Tarasov et al., 2021). In addition, EVs can be further engineered by targeting ligands, stimuli-responsive elements, and immune evasion properties, et al. (Armstrong et al., 2017), which will maximize their drug delivery potential. For the treatment of melanoma, EVs as vehicles can load a variety of drugs or cargoes, such as conventional chemotherapy drugs, polypeptides, oligonucleotides, amino acids, immunostimulants, et al. As a result, due to their intrinsic and modified properties obtained by vesicle engineering, EVs offer promising prospects for melanoma therapy by its self-biomolecules (e.g., micro RNAs) or the improved delivery of other therapeutic agents. Herein, we summarize the recent advances of EVs as both therapeutics themself and delivery vehicles in the treatment of melanoma (Table 1). The prospect and obstacles related to their clinical translation will also be discussed.

**TABLE 1:** Exosome based therapeutic strategies for melanoma.

EVs (source)	Cargo/drug	*In vitro*/*in vivo* models	Results	Ref
EVs based endogenous miRNAs				
A375 and SK-MEL-28	miR-191	HEMa-LP and NHEM-c cells	Promote epithelial-to-mesenchymal transition through MAPK signaling pathway	Xiao et al. (2016)
A375 and SK-MEL-28	miR-106b-5p	HEMa-LP/A375-bearing metastasis mice	Activate the ERK pathway and induces the EMT of melanocytes; Promoted melanoma metastasis	Luan et al. (2021)
B16 cells, B16.F10	miR-155	NIH/3T3 cells/C57BL/6 mice	Induce the proangiogenic switch of CAFs by promoting the expression of proangiogenic factors *via* SOCS1/JAK2/STAT3 signaling pathway	Zhou et al. (2018)
Six melanoma cell lines	miR-155 and miR-210	HADF	Create a pre-metastatic niche that promotes the development of metastasis by promoting glycolysis and inhibiting OXPHOS in HADF.	Shu et al. (2018)
Commercial milk	Bovine-Specific miR-2478	B16.F10, MNT-1 cells	Decrease melanin contents, tyrosinase activity and the expression of melanogenesis-related genes in melanoma cells; Decrease melanogenesis through the Akt-GSK3β signal pathway	Bae and Kim, (2021)
A375	miR-494	A375, WM35/A375-bearing mice	Suppress proliferation and metastasis of melanoma cells and tumors by blockage of miR-494 transfer	Li et al. (2019)
EVs based chemotherapy				
B16.F10	Dox	B16.F10/B16.F10 bearing mice	Higher inhibitory effects on the proliferation and a stronger antitumor efficacy in PEG-EV-Dox group	Patras et al. (2022)
B16.F10	Dox	B16.F10/B16.F10 C57BL/6 mice	Strong antiproliferative effect on tumor cells co-cultured with TAMs by administration of IL-13-LCL-SIM and PEG-EV-Dox	Negrea et al. (2022)
B16.F10	Dox	B16.F10/B16.F10 bearing mice	Therapeutic activity of Dox exosomes was enhanced by an active targeting ASL; High intratumorally and minimal systemic toxicity	Kang et al. (2020)
Raw264.7 cells	Triptolide	A375/A375-bearing BALB/c nude mice	Improved tumor targetability, enhanced cellular uptake, inhibited proliferation, invasion, and migration of melanoma and reduced the toxicity of Triptolide	Jiang et al. (2021)
Macrophages	Acridine Orange	Me 30,966	Longer retention and increased cytotoxicity	Iessi et al. (2017)
EVs based immunotherapy				
Ginseng	none	B16.F10/B16.F10-bearing C57BL/6 mice	GDNPs can alter M2 macrophages polarization, which contributes to an antitumor response both *in vitro* and *in vivo*	Cao et al. (2019)
NK-92MI cells	none	B16.F10/B16.F10-bearing C57BL/6 mice	Induce B16.F10 cell apoptosis *in vitro*; Inhibit tumor growth and tumor volume reduction *in vivo*	Zhu et al. (2017)
M1-like macrophages	none	B16-bearing C57BL/6 mice	Accumulate in both lymph nodes and tumors of xenograft mice; Prime T cell activation; Induce tumor regression and extend survival in primary mouse models	Wang et al. (2021)
HEK 293T cells	PD-1 receptors/1-methyl-tryptophan	B16.F10/B16.F10-bearing C57BL/6 mice	PD-1 engineered cellular nanovesicles (PD-1 NVs) enhance antitumor responses by disrupting the PD-1/PD-L1 immune inhibitory axis. 1-methyl-tryptophan synergistically disrupt immune tolerance pathway	Zhang et al. (2018)
Bone marrow derived MSC	TRAIL	B16F0/B16F0-bearing C57BL/6 mice	Induce B16.F0 cell apoptosis *in vitro*. Reduce tumor volume *in vivo*	Shamili et al. (2018)
Raw 264.7	TGFβRI kinase inhibitor/TLR-7/8 resiquimod	B16.F10/B16.F10-bearing mice	Reduce the migration of B16.F10; Trigger the release of proinflammatory cytokines from stimulated macrophages and dendritic cells; Reduced tumor growth and improved survival rate at low doses by the combination therapy of R848/EXOs and SD-208/EXOs	Lee et al. (2022)
B16BL6	CpG-DNA	B16BL6-bearing mice	Selectively taken up by APCs; Activated dendritic cells *in vitro*; Prolonged tissue residence and increased the immune responses	Matsumoto et al. (2019)
B16BL6	CpG DNA	B16BL6-bearing mice	CpG-SAV-exo activated DC2.4 cells and enhanced tumor antigen presentation capacity; Immunization with CpG-SAV-exo exhibited stronger antitumor effects *in vivo* than simple co-administration of exosomes and CpG DNA.	Morishita et al. (2016)
Autologous monocyte derived DC	MAGE 3 peptides	Phase I clinical trial in stage III/IV melanoma patients	The feasibility of large-scale exosome production and the safety of exosome administration were confirmed	Escudier et al. (2005)
EVs based photothermal therapy				
B16.F10, DC	AuNP	B16.F10 cells/Murine melanoma model	Efficiently kill B16.F10 cells with laser irradiation in term of cytotoxicity and therapeutic efficacy; Significant tumor growth suppression with the inhibitory rate of 96.7% antitumor effect	Zhang et al. (2019b)
DC2.4	Dox and AuNP	B16.F10 cells/B16.F10-bearing C57BL/6 mice	Improved cellular internalization, controlled drug release, enhanced antitumor efficacy with tumor inhibitory rate up to 98.6% and reduced side effects	Zhang et al. (2019a)
B16.F10	Fe^3+^ and exosome inhibitor (GW4869)	A resistant B16.F10 tumor mice	Superior near-infrared II fluorescence/photoacoustic imaging tracking performance for a precise PTT; Elieved exosomal silencing on DC maturation; Evitalized T cells and enhanced the ferroptosis	Xie et al. (2022)
EVs based radiotherapy				
B16.F10	^125^I-TA99	B16.F10 cells/B16.F10-bearing C57BL/6 mice	About 30–40% of melanoma cells were killed by CM *in vitro*; sEVs cytotoxicity could not be detected *in vivo*	Karam et al. (2021)
B16.F10 irradiated with 137Cs γ-rays	Calreticulin, HSP70, TSG101	B16.F10-bearing C57BL/6 mice	Lead to tumor growth delay in a NK cell dependent manner by activating APCs and NK cells	Jella et al. (2020)
Reradiated umbilical-cord stromal stem cells	ANXA1, ANAX2	A375 -bearing NOD/SCID-gammac (NSG) mice	Improved tumor cell loss rates; Increased the mice surviving time; Decreased metastatic foci	de Araujo Farias et al. (2018)

### EVs based endogenous miRNAs for melanoma treatment

Primarily, EVs enable intercellular communications *via* a wide range of endogenous bioactive molecules, such as RNAs, proteins, carbohydrates, and lipids. When it comes to the treatment of melanoma, miRNAs are often mentioned for their well-known roles in tumorigenesis. (Ingenito et al., 2019). In this way, miRNAs may offer novel tumor therapeutic targets in melanoma therapies. Here, we discussed recent studies exploring the potentials of exosomal miRNAs for melanoma treatment.

It is generally known that the metastasis of melanoma involves multiple steps, one of which is epithelial-to-mesenchymal transition (EMT). Compared with most of the epithelial tissues, melanocytes express large amounts of EMT-inducing transcription factors, an intrinsic factor predisposing melanoma to high-grade metastatic property. (Xiao et al., 2016). McMasters et al. reported that miR-191 derived from two human malignant melanoma cell lines, A375 and SK-MEL-28, could promote EMT in primary melanocytes through the mitogen-activated protein kinase (MAPK) signaling pathway. (Xiao et al., 2016). Similarly, Wang et al. found that miR-106b-5p could induce the EMT of human epidermal melanocytes (HEMa-LP) and promote melanoma metastasis in mice model with pulmonary tumor metastasis. (Luan et al., 2021). Novel strategies targeting EMT may offer as new approaches for the treatment of melanoma, in which relevant miRNAs may be recognized as potential therapeutic targets.

Besides, exosomal miRNAs can affect the tumor microenvironment in the occurrence and development of melanoma. It is reported that miR-155 may be a potential target for controlling melanoma angiogenesis by switching the expression of proangiogenic factors in cancer-associated fibroblasts (CAFs) *via* the SOCS1/JAK2/STAT3 signaling pathway. (Zhou et al., 2018) (Figure 1B). Moreover, Ernstof *et al.* demonstrated that exosomal miR-155/miR-210 modulates stromal cell metabolism and may contribute to a pre-metastatic niche. (Shu et al., 2018). When human adult dermal fibroblasts (HADF) were exposed to human melanoma-derived exosomes (HMEX), an increase in aerobic glycolysis and a decrease in oxidative phosphorylation (OXPHOS) of HADF were observed. Exosomes derived from milk were also developed for the therapy of melanoma. For example, Kim’s research supports that the miR-2478 derived from milk exosomes can regulate melanogenesis by targeting Rap1a (a target gene of miR-2478) in melanoma cells through the AKT-GSK3β signal pathway. (Bae and Kim, 2021).

In another study by Zhou *et al.* (Li et al., 2019), they investigated the roles of miR-494 in melanoma progression. Their results showed that the proliferation and metastasis of melanoma cells were suppressed by inhibiting the release of exosomes, thus blocking the transfer of miR-494. Meanwhile, *in vivo* studies further confirmed that tumor growth and metastasis were suppressed by increasing miR-494 accumulation after the depletion of rab27a (GTPase, a well-known inhibitor for exosome release). It implies that blocking the exosomal transfer of miR-494 may offer another new approach to developing miRNA-based therapies for melanoma.

### EVs based chemotherapy for melanoma

Chemotherapy is a widely accepted palliative therapy for stage IV metastatic melanoma. (Atkins et al., 2008). Dacarbazine as a single chemotherapeutic agent is the most widely used for the treatment of metastatic melanoma. Unfortunately, most responses to this agent are transient because only 1%–2% of patients achieved a durable long-term response to chemotherapy (Eisen, 2003), not to mention the common and unacceptable reactions to chemotherapy drugs. Compared with chemotherapeutic agents alone, relevant studies demonstrated a survival benefit from EVs-based chemotherapy, including efficacy improvement, reduced off-target effects, lower cytotoxic effects on normal cells et al. (Elsharkasy et al., 2020) It has been shown in many clinical trials that the decreased responsiveness of melanomas to doxorubicin (Dox) causes therapeutic inefficacy. (Fink et al., 2004; Smylie et al., 2007). However, Patras et al*.* exemplified that EVs loaded with Dox and modified with polyethylene glycol (PEG) moieties (PEG-EV-Dox) exerted much higher inhibitory effects on the proliferation of mouse melanoma cells, B16F10. The following *in vivo* experiments in B16.F10 melanoma-bearing mice also revealed that the PEG-EV-Dox showed a more robust antitumor efficacy. PEG-EV-Dox ensures prolonged systemic circulation time, higher accumulation of Dox, and more powerful tumor targeting potential. (Patras et al., 2022) (Figure 1C). Their latest study proposed a combination therapy with simvastatin incorporated in IL-13-functionalized long-circulating liposomes (IL-13-LCL-SIM) based on PEG-EV-Dox to selectively target both tumor-associated macrophages and melanoma cells. (Negrea et al., 2022). The results demonstrated that sequential administration of IL-13-LCL-SIM and PEG-EV-Dox had the most potent antiproliferative effect on melanoma cells.

In this regard, another researcher developed a more complex platform focusing on delivering Dox to melanoma with an active targeting modality, which composed of membrane anchor (BODIPY)-spacer (PEG)-targeting ligands (cyclic RGD peptide), namely, AExs. Dox encapsulated AExs (dAExs) delivered significantly enhanced anticancer activity, exhibiting the lowest half-maximal inhibitory concentrations (IC50) of 1.606 ± 0.255 μM compared to free Dox groups (9.852 ± 1.058 μM). Moreover, tumor volume and body weight of the dAEx group were exhibited significant suppression of melanoma growth, indicating the enhanced therapeutic activity of Dox through targeted and improved delivery *via* modified EVs. (Kang et al., 2020). These results are consistent with another similar example of engineered exosomes. (Jiang et al., 2021). By contrast, they chose tumor necrosis factor (TNF)-related apoptosis-inducing ligand (TRAIL) as an engineering tool due to its property of inducing extrinsic apoptosis in most cancerous cells. In addition, the drug delivered by exosomes is triptolide (TPL), which possesses diversified pharmacological activities, including antineoplastic. Similarly, Elisabetta et al*.* reported a system of macrophage-derived exosomes loaded tumoricidal drug, acridine orange (AO), showing greater antitumor effectiveness against metastatic melanoma cells than free AO. (Iessi et al., 2017).

### EVs based immunotherapy for melanoma

Traditional immunotherapy for metastatic melanoma is mainly divided into the following five categories: cytokines such as interleukin 2 (IL-2) and α interferon (IFN-α), tumor vaccine based on melanoma antigen or DC, oncolytic virus, T cell adoptive therapy and immune checkpoint inhibitors (ICIs). (Zhang and Zhang, 2020). In recent years, immunotherapy represented by immune checkpoint blockade (ICB) has dramatically improved the prognosis of patients with metastatic melanoma. However, melanoma immunotherapy still faces some challenges such as drug resistance and immune-related adverse effects (irAE). (Jenkins and Fisher, 2021). The combination of exosomes and immunotherapy has the potential not only to change the drug delivery modes of traditional immunotherapy, optimize pharmacokinetics and reduce the occurrence of irAE, but also help to accurately target the tumor immunosuppressive microenvironment and enhance the efficacy of immunotherapy. (Irvine and Dane, 2020). Up to now, various studies have shown that EVs-based immunotherapy can significantly inhibit tumor progression by multiple mechanisms such as more robust tumor immune response and immune memory etc*.* (Shi and Lammers, 2019)

A series of studies have shown that exosomes without extra drugs could play an immunotherapeutic role in melanoma. Zhu *et al.* found that exosomes derived from NK cells can induce B16F10 cells apoptosis and inhibit the growth of melanoma xenografts. The mechanism may be related to Fas-L and TNF-α in exosomes from NK cells. (Zhu et al., 2017). Similarly, another study revealed that ginseng-derived nanoparticles (GDNPs) could alter macrophage M2 polarization, which contributes to an antitumor response both *in vitro* and *in vivo*. (Cao et al., 2019). Moreover, Ma et al*.* developed chimeric exosomes with the function of dual targeting of lymph nodes and tumor tissues, which activated the immune response in lymph nodes and improved the tumor immune microenvironment, showing significantly inhibited tumor progression in melanoma mice models *via* synergistic mechanism. (Wang et al., 2021).

Apart from exogenous drug-free exosomes, the following findings are compelling proofs of the excellent antitumor effects toward melanoma elicited with engineered exosomes. Zhang and colleagues reported engineered EVs displaying PD-1 on the surface, disrupting the immunosuppressive PD-1/PD-L1 axis by shielding PD-L1 on melanoma cancer cells. (Zhang et al., 2018). Furthermore, an inhibitor of indoleamine 2,3-dioxygenase was loaded into the PD-1 modified EVs, which inhibited the immunosuppressive pathway, leading to increased tumor infiltration of CD8^+^ T cells and tumor regression. Similar results were observed by Abnous et al*.*, whilst the engineered exosome strategy is transfecting bone marrow-derived MSCs with plasmid encoding TRAIL, a novel agent for cancer treatment acting as tumor necrosis factor-related apoptosis-inducing ligand. (Shamili et al., 2018) (Figure 1D).

Combination therapy of the transforming growth factor-β receptor I (TGFβRI) kinase inhibitor SD-208 and a toll-like receptor (TLR)-7/8 agonist resiquimod (R848) was examined along with serum-derived exosomes (EXOs) as versatile carriers. *In vitro*, SD-208/EXOs and R848/EXOs reduced the migration of B16F10 cells and triggered the release of proinflammatory cytokines from stimulated macrophages and dendritic cells, respectively. In addition, the combination therapy of R848/EXOs and SD-208/EXOs reduced tumor growth and improved survival rate at low doses in the B16F10 tumor xenograft model. (Lee et al., 2022).

Besides, tumor-cell derived small extracellular vesicles (sEV) combined with immunostimulatory adjuvants may serve as a promising tumor vaccine through the induction of the cytotoxic T cell response. Takakura et al*.* proposed an efficient delivery system based on tumor-cell derived exosomes that contain endogenous tumor antigens and immunostimulatory CpG DNA. Murine melanoma B16BL6 cells were transfected with a plasmid vector encoding a fusion streptavidin (SAV, a protein that binds to biotin with high affinity)-lactadherin (LA, an exosome-tropic protein) protein, yielding genetically engineered SAV-LA-expressing exosomes (SAV-Exo). Consequently, treatment with CpG-SAV-Exo effectively activated mouse dendritic cells (DC2.4 cells) and enhanced tumor antigen presentation capacity. Immunization with CpG-SAV- Exo exhibited stronger antitumor effects *in vivo* than simple co-administration of exosomes and CpG DNA. (Morishita et al., 2016). The authors also constructed a CpG-DNA-anchored superstructure in which sEVs were assembled to achieve prolonged tissue residence and the selective uptake by dendritic cells. (Matsumoto et al., 2019).

As early as 2005, exosomes have been reported for clinical immunotherapy of melanoma. Escudier et al*.* conducted phase I clinical trials for patients with stage III/IV melanoma immunotherapy using exosomes derived from dendritic cells with MAGE3 polypeptide. The results showed that its large-scale application in clinical is safe and feasible. (Escudier et al., 2005).

### EVs based photothermal therapy for melanoma

Photothermal therapy (PTT) is a treatment method composed of multiple procedures, including preparation of biomaterials with high photothermal conversion efficiency, the irradiation of external light sources (generally near-infrared light), temperature rising of tumor tissue, and killing of tumor cells etc*.* (Tong et al., 2007) Although PTT is mainly focused on local tumor treatments, these treatments could cause tumor-specific immune responses. This immune response is mainly regulated by tumor antigens and heat shock proteins released by dead tumor cells, which can be captured by antigen-presenting cells, with a consequence of activating the immune system to attack distal tumor cells. In other words, the photothermal effect could also be expected to treat distal sites, even metastases. (Ito et al., 2006; Greten and Korangy, 2010). For example, Zhang et al. (Zhang et al., 2019a) developed a novel immunological gold nanoparticle (AuNP) for combinatorial PTT and immunotherapy against melanoma (Figure 1E). This nanoparticle was intracellularly generated, followed by exocytosis from B16.F10 cells with retained tumor antigens (AuNP@B16.F10), then further internalized by DCs and secreted as DCs derived vesicles (AuNP@DCB16.F10). The *in vitro* cytotoxicity and anti-cancer assays show that AuNP@DCB16.F10 can efficiently kill tumor cells with laser irradiation, while nanoparticles or lasers alone cannot. In the *in vivo* assay, AuNP@DCB16.F10 + NIR (near infrared) exhibited significant tumor growth suppression with an inhibitory rate of 96.7%. Mechanistically, under NIR irradiation, AuNP@ DCB16.F10 generated heat and caused apoptosis of the tumor cells, which further enhanced antitumor immunity as a prominent immunological nanoplatform. Others also reported a novel metal nanostructure named EVdox@AuNP for combinatorial chemo-photothermal therapy. (Zhang et al., 2019b). EVdox@AuNP, consisting of self-grown gold nanoparticles, EVs, and Dox, demonstrated improved cellular internalization, controlled drug release, and more importantly, enhanced antitumor efficacy and better biocompatibility. Furthermore, Dai et al*.* (Xie et al., 2022) developed a new multifunctional nanomedicine named phototheranostic metal-phenolic networks (PFG MPNs) by integration of semiconductor polymers, ferroptosis inducer (Fe^3+^), and exosomes inhibitor (GW4869). The PFG MPNs demonstrated superior near-infrared II fluorescence/photoacoustic imaging performance for PTT. GW4869 mediated PD-L1 based exosomal inhibition revitalized T cells and enhanced the ferroptosis. This synergy strategy of PTT with anti-exosomal PD-L1 enhanced ferroptosis evoked potent antitumor immunity in B16.F10 tumors and immunological memory against metastatic tumors in lymph nodes.

### EVs based radiotherapy for melanoma

For melanoma therapy, radiotherapy (RT) has been recognized as a tumor-localized treatment aimed at local control or palliation in the past decades. (Barton et al., 2014). Besides, considering the tolerance of the peritumoral tissues, the dose limits often preclude the application of curative radiation. (Dietrich et al., 2015). However, several recent studies revealed that EVs-based radiotherapy may provide a novel recognition. In a sense, EVs-based radiotherapy may not only be a successful regional treatment but also a promising systemic strategy for melanoma. In the study of Karam *et al.* (Karam et al., 2021)*,* they reported preliminary results on the role of small extracellular vesicles (sEVs) as mediators of bystander and systemic effects in melanoma cells exposed to Auger RIT (radioimmunotherapy) or X-rays (Figure 1F). sEVs isolated from conditioned medium (CM) of Auger-RIT exposed cells are associated with about 30–40% bystander cytotoxic effects. They also observed that progressively, cytoplasmic dsDNA increased in B16.F10 cells during Auger-RIT and was enriched in sEVs. However, sEV-based *in vivo* studies showed no therapeutic effects. The authors suspected that sEVs collection time might impact the nature of sEVs, which should be further investigated. Similarly, Kishore et al*.* (Jella et al., 2020) provide novel mechanistic insights into the post-irradiation non-targeted effects of radiotherapy. According to their results, irradiation triggers the release of tumor-cell exosomes (RT-TEX) from B16.F10 cells, which contain several DAMPs (damage-associated molecular pattern) that activate antigen-presenting cell (APCs) and NK cells and delay tumor growth. However, since the ability of RT-TEX to activate NK cells has so far been investigated only in the context of *in vivo* tumor milieu, the mechanism underlying direct or indirect influence of exosomes on NK cells remains unknown. Next, Almodóvar *et al.* (de Araujo Farias et al., 2018) presented preclinical therapeutic data combining RT with MSC (mesenchymal stem cells) therapy. Their study aimed to investigate the role of exosomes derived from irradiated MSCs in delaying melanoma growth and metastasis after treatment with MSC + RT. They found that the tumor cell loss rates were: 44.4% and 12.1%, respectively, after treatment with the combination of MSC and RT and for exclusive RT. Moreover, the number of metastatic foci *in vivo* treated with MSC + RT was 60%, less than the control group, which shows that exosomes derived from MSCs, combined with radiotherapy, enhanced radiation effects and possessed superior melanoma suppressive effect.

## Conclusion and perspectives

The components derived from exosomes themselves can affect the recipient cells’ physiological processes and influence the melanoma microenvironment *via* cell-to-cell communications. More importantly, due to their physicochemical and biological properties, exosomes are considered very promising drug/cargo delivery vehicles for the treatment of melanoma, especially with the assistance of advanced bioengineering techniques, including surface modification, various loading techniques, nucleic acid delivery techniques, etc*.* In other words, modification strategies offer the promising prospect of extending the therapeutic capability of EVs beyond their natural functions. (Miles et al., 2020). In this minireview, we have briefly classified the research progress of exosomes in melanoma treatment according to the different drugs/cargo, including chemotherapy, immunotherapy, photothermal therapy, radiotherapy, and their synergic therapy.

Despite the prospects mentioned above, the obstacles to applying exosomes in clinical practice cannot be ignored. To begin with, the lack of standardized/scalable EV isolation techniques, storage methods, and appropriate quality controls have hampered further translation of EVs. (Li et al., 2020). Furthermore, the biosafety of EVs-based nanomedicines also needs to be addressed. (Walker et al., 2019). More insights into the issues mentioned above will likely pave the way for unlocking EVs’ full potential and broader applications of promising EV-based melanoma therapies in the near future.

## References

[B1] Ahmadi M. Rezaie J. (2020). Tumor cells derived-exosomes as angiogenenic agents: Possible therapeutic implications. J. Transl. Med. 18 (1), 249. 10.1186/s12967-020-02426-5 32571337 PMC7310379

[B2] Armstrong J. P. Holme M. N. Stevens M. M. (2017). Re-engineering extracellular vesicles as smart nanoscale therapeutics. ACS Nano 11 (1), 69–83. 10.1021/acsnano.6b07607 28068069 PMC5604727

[B3] Atkins M. B. Hsu J. Lee S. Cohen G. I. Flaherty L. E. Sosman J. A. (2008). Eastern cooperative oncology, GPhase III trial comparing concurrent biochemotherapy with cisplatin, vinblastine, dacarbazine, interleukin-2, and interferon alfa-2b with cisplatin, vinblastine, and dacarbazine alone in patients with metastatic malignant melanoma (E3695): A trial coordinated by the eastern cooperative oncology group. J. Clin. Oncol. 26 (35), 5748–5754. 10.1200/JCO.2008.17.5448 19001327 PMC2645104

[B4] Bae I. S. Kim S. H. (2021). Milk exosome-derived MicroRNA-2478 suppresses melanogenesis through the akt-gsk3β pathway. Cells 10 (11), 2848. 10.3390/cells10112848 34831071 PMC8616206

[B5] Bandarchi B. Ma L. Navab R. Seth A. Rasty G. (2010). From melanocyte to metastatic malignant melanoma. Dermatol. Res. Pract. 2010, 1–8. 10.1155/2010/583748 PMC2948895 20936153

[B6] Barton M. B. Jacob S. Shafiq J. Wong K. Thompson S. R. Hanna T. P. (2014). Estimating the demand for radiotherapy from the evidence: A review of changes from 2003 to 2012. Radiother. Oncol. 112 (1), 140–144. 10.1016/j.radonc.2014.03.024 24833561

[B7] Cao M. Yan H. Han X. Weng L. Wei Q. Sun X. (2019). Ginseng-derived nanoparticles alter macrophage polarization to inhibit melanoma growth. J. Immunother. Cancer 7 (1), 326. 10.1186/s40425-019-0817-4 31775862 PMC6882204

[B8] Chen H. Hou K. Yu J. Wang L. Chen X. (2022). Nanoparticle-based combination therapy for melanoma. Front. Oncol. 12, 928797. 10.3389/fonc.2022.928797 35837089 PMC9273962

[B9] de Araujo Farias V. O'Valle F. Serrano-Saenz S. Anderson P. Andres E. Lopez-Penalver J. (2018). Exosomes derived from mesenchymal stem cells enhance radiotherapy-induced cell death in tumor and metastatic tumor foci. Mol. Cancer 17 (1), 122. 10.1186/s12943-018-0867-0 30111323 PMC6094906

[B10] Dietrich A. Koi L. Zophel K. Sihver W. Kotzerke J. Baumann M. (2015). Improving external beam radiotherapy by combination with internal irradiation. Br. J. Radiol. 88 (1051), 20150042. 10.1259/bjr.20150042 25782328 PMC4628530

[B11] Du T. Yang C. L. Ge M. R. Liu Y. Zhang P. Li H. (2020). M1 macrophage derived exosomes aggravate experimental autoimmune neuritis via modulating Th1 response. Front. Immunol. 11, 1603. 10.3389/fimmu.2020.01603 32793234 PMC7390899

[B12] Eisen M. B. L. T. G. Eisen T. G. (2003). Systemic chemotherapy in the treatment of malignant melanoma. Expert Opin. Pharmacother. 4 (12), 2205–2211. 10.1517/14656566.4.12.2205 14640919

[B13] Elsharkasy O. M. Nordin J. Z. Hagey D. W. de Jong O. G. Schiffelers R. M. Andaloussi S. E. (2020). Extracellular vesicles as drug delivery systems: Why and how? Adv. Drug Deliv. Rev. 159, 332–343. 10.1016/j.addr.2020.04.004 32305351

[B14] Escudier B. Dorval T. Chaput N. Andre F. Caby M. P. Novault S. (2005). Vaccination of metastatic melanoma patients with autologous dendritic cell (DC) derived-exosomes: Results of thefirst phase I clinical trial. J. Transl. Med. 3 (1), 10. 10.1186/1479-5876-3-10 15740633 PMC554765

[B15] Fink W. Zimpfer-Rechner C. Thoelke A. Figl R. Kaatz M. Ugurel S. (2004). Clinical phase II study of pegylated liposomal doxorubicin as second-line treatment in disseminated melanoma. Oncol. Res. Treat. 27 (6), 540–544. 10.1159/000081335 15591712

[B16] Fu C. Zhou L. Mi Q. S. Jiang A. (2022). Plasmacytoid dendritic cells and cancer immunotherapy. Cells 11 (2), 222. 10.3390/cells11020222 35053338 PMC8773673

[B17] Garbe C. Eigentler T. K. Keilholz U. Hauschild A. Kirkwood J. M. (2011). Systematic review of medical treatment in melanoma: Current status and future prospects. Oncologist 16 (1), 5–24. 10.1634/theoncologist.2010-0190 21212434 PMC3228046

[B18] Greten T. F. Korangy F. (2010). Radiofrequency ablation for the treatment of HCC--maybe much more than simple tumor destruction? J. Hepatology 53 (4), 775–776. 10.1016/j.jhep.2010.05.008 20619917

[B19] Herrmann I. K. Wood M. J. A. Fuhrmann G. (2021). Extracellular vesicles as a next-generation drug delivery platform. Nat. Nanotechnol. 16 (7), 748–759. 10.1038/s41565-021-00931-2 34211166

[B20] Iessi E. Logozzi M. Lugini L. Azzarito T. Federici C. Spugnini E. P. (2017). Acridine orange/exosomes increase the delivery and the effectiveness of acridine orange in human melanoma cells: A new prototype for theranostics of tumors. J. Enzyme Inhib. Med. Chem. 32 (1), 648–657. 10.1080/14756366.2017.1292263 28262028 PMC6010124

[B21] Ingenito F. Roscigno G. Affinito A. Nuzzo S. Scognamiglio I. Quintavalle C. (2019). The role of exo-miRNAs in cancer: A focus on therapeutic and diagnostic applications. Int. J. Mol. Sci. 20 (19), 4687. 10.3390/ijms20194687 31546654 PMC6801421

[B22] Irvine D. J. Dane E. L. (2020). Enhancing cancer immunotherapy with nanomedicine. Nat. Rev. Immunol. 20 (5), 321–334. 10.1038/s41577-019-0269-6 32005979 PMC7536618

[B23] Ito A. Honda H. Kobayashi T. (2006). Cancer immunotherapy based on intracellular hyperthermia using magnetite nanoparticles: A novel concept of "heat-controlled necrosis" with heat shock protein expression. Cancer Immunol. Immunother. 55 (3), 320–328. 10.1007/s00262-005-0049-y 16133113 PMC11030207

[B24] Jella K. K. Nasti T. H. Li Z. Lawson D. H. Switchenko J. M. Ahmed R. (2020). Exosome-containing preparations from postirradiated mouse melanoma cells delay melanoma growth *in vivo* by a natural killer cell-dependent mechanism. Int. J. Radiat. Oncology*Biology*Physics 108 (1), 104–114. 10.1016/j.ijrobp.2020.06.016 PMC7751005 32561502

[B25] Jenkins R. W. Fisher D. E. (2021). Treatment of advanced melanoma in 2020 and beyond. J. Invest. Dermatol. 141 (1), 23–31. 10.1016/j.jid.2020.03.943 32268150 PMC7541692

[B26] Jiang L. Gu Y. Du Y. Tang X. Wu X. Liu J. (2021). Engineering exosomes endowed with targeted delivery of triptolide for malignant melanoma therapy. ACS Appl. Mat. Interfaces 13 (36), 42411–42428. 10.1021/acsami.1c10325 34464081

[B27] Kang C. Han P. Lee J. S. Lee D. Kim D. (2020). Anchor, spacer, and ligand-modified engineered exosomes for trackable targeted therapy. Bioconjug. Chem. 31 (11), 2541–2552. 10.1021/acs.bioconjchem.0c00483 33115231

[B28] Karam J. Constanzo J. Pichard A. Gros L. Chopineau J. Morille M. (2021). Rapid communication: Insights into the role of extracellular vesicles during auger radioimmunotherapy. Int. J. Radiat. Biol. 97, 1–10. 10.1080/09553002.2021.1955999 34270378

[B29] Lee J. H. Song J. Kim I. G. You G. Kim H. Ahn J. H. (2022). Exosome-mediated delivery of transforming growth factor-beta receptor 1 kinase inhibitors and toll-like receptor 7/8 agonists for combination therapy of tumors. Acta Biomater. 141, 354–363. 10.1016/j.actbio.2022.01.005 35007784

[B30] Li C. Donninger H. Eaton J. Yaddanapudi K. (2020). Regulatory role of immune cell-derived extracellular vesicles in cancer: The message is in the envelope. Front. Immunol. 11, 1525. 10.3389/fimmu.2020.01525 32765528 PMC7378739

[B31] Li J. Chen J. Wang S. Li P. Zheng C. Zhou X. (2019). Blockage of transferred exosome-shuttled miR-494 inhibits melanoma growth and metastasis. J. Cell. Physiol. 234, 15763–15774. 10.1002/jcp.28234 30723916

[B32] Luan W. Ding Y. Xi H. Ruan H. Lu F. Ma S. (2021). Exosomal miR-106b-5p derived from melanoma cell promotes primary melanocytes epithelial-mesenchymal transition through targeting EphA4. J. Exp. Clin. Cancer Res. 40 (1), 107. 10.1186/s13046-021-01906-w 33741023 PMC7980627

[B33] Matsumoto A. Takahashi Y. Ariizumi R. Nishikawa M. Takakura Y. (2019). Development of DNA-anchored assembly of small extracellular vesicle for efficient antigen delivery to antigen presenting cells. Biomaterials 225, 119518. 10.1016/j.biomaterials.2019.119518 31586864

[B34] Miles J. A. Caobi A. Raymond A. D. Nair M. (2020). Bioengineered exosomal extracellular vesicles in cancer therapeutics. Crit. Rev. Biomed. Eng. 48 (3), 177–187. 10.1615/critrevbiomedeng.2020034847 33389895 PMC11102805

[B35] Morishita M. Takahashi Y. Matsumoto A. Nishikawa M. Takakura Y. (2016). Exosome-based tumor antigens-adjuvant co-delivery utilizing genetically engineered tumor cell-derived exosomes with immunostimulatory CpG DNA. Biomaterials 111, 55–65. 10.1016/j.biomaterials.2016.09.031 27723556

[B36] Munich S. Sobo-Vujanovic A. Buchser W. J. Beer-Stolz D. Vujanovic N. L. (2012). Dendritic cell exosomes directly kill tumor cells and activate natural killer cells via TNF superfamily ligands. Oncoimmunology 1 (7), 1074–1083. 10.4161/onci.20897 23170255 PMC3494621

[B37] Negrea G. Rauca V. F. Meszaros M. S. Patras L. Luput L. Licarete E. (2022). Active tumor-targeting nano-formulations containing simvastatin and doxorubicin inhibit melanoma growth and angiogenesis. Front. Pharmacol. 13, 870347. 10.3389/fphar.2022.870347 35450036 PMC9016200

[B38] Patras L. Ionescu A. E. Munteanu C. Hajdu R. Kosa A. Porfire A. (2022). Trojan horse treatment based on PEG-coated extracellular vesicles to deliver doxorubicin to melanoma *in vitro* and *in vivo* . Cancer Biol. Ther. 23 (1), 1–16. 10.1080/15384047.2021.2003656 PMC8812761 34964693

[B39] Shamili F. H. Bayegi H. R. Salmasi Z. Sadri K. Mahmoudi M. Kalantari M. (2018). Exosomes derived from TRAIL-engineered mesenchymal stem cells with effective anti-tumor activity in a mouse melanoma model. Int. J. Pharm. X. 549 (1-2), 218–229. 10.1016/j.ijpharm.2018.07.067 30075248

[B40] Shi Y. Lammers T. (2019). Combining nanomedicine and immunotherapy. Acc. Chem. Res. 52 (6), 1543–1554. 10.1021/acs.accounts.9b00148 31120725 PMC7115879

[B41] Shu S. Yang Y. Allen C. L. Maguire O. Minderman H. Sen A. (2018). Metabolic reprogramming of stromal fibroblasts by melanoma exosome microRNA favours a pre-metastatic microenvironment. Sci. Rep. 8 (1), 12905. 10.1038/s41598-018-31323-7 30150674 PMC6110845

[B42] Smylie M. G. Wong R. Mihalcioiu C. Lee C. Pouliot J. F. (2007). A phase II, open label, monotherapy study of liposomal doxorubicin in patients with metastatic malignant melanoma. Invest. New Drugs 25 (2), 155–159. 10.1007/s10637-006-9002-y 16957835

[B43] Sun W. Luo J. D. Jiang H. Duan D. D. (2018). Tumor exosomes: A double-edged sword in cancer therapy. Acta Pharmacol. Sin. 39 (4), 534–541. 10.1038/aps.2018.17 29542685 PMC5888693

[B44] Tarasov V. V. Svistunov A. A. Chubarev V. N. Dostdar S. A. Sokolov A. V. Brzecka A. (2021). Extracellular vesicles in cancer nanomedicine. Semin. Cancer Biol. 69, 212–225. 10.1016/j.semcancer.2019.08.017 31421263

[B45] Tong L. Zhao Y. Huff T. B. Hansen M. N. Wei A. Cheng J. X. (2007). Gold nanorods mediate tumor cell death by compromising membrane integrity. Adv. Mat. 19, 3136–3141. 10.1002/adma.200701974 PMC2584614 19020672

[B46] van der Meel R. Sulheim E. Shi Y. Kiessling F. Mulder W. J. M. Lammers T. (2019). Smart cancer nanomedicine. Nat. Nanotechnol. 14 (11), 1007–1017. 10.1038/s41565-019-0567-y 31695150 PMC7227032

[B47] Walker S. Busatto S. Pham A. Tian M. Suh A. Carson K. (2019). Extracellular vesicle-based drug delivery systems for cancer treatment. Theranostics 9 (26), 8001–8017. 10.7150/thno.37097 31754377 PMC6857056

[B48] Wang C. Huang X. Wu Y. Wang J. Li F. Guo G. (2020). Tumor cell-associated exosomes robustly elicit anti-tumor immune responses through modulating dendritic cell vaccines in lung tumor. Int. J. Biol. Sci. 16 (4), 633–643. 10.7150/ijbs.38414 32025211 PMC6990923

[B49] Wang S. Li F. Ye T. Wang J. Lyu C. Qing S. (2021). Macrophage-tumor chimeric exosomes accumulate in lymph node and tumor to activate the immune response and the tumor microenvironment. Sci. Transl. Med. 13, eabb6981. 10.1126/scitranslmed.abb6981 34644149

[B50] Witwer K. W. Thery C. (2019). Extracellular vesicles or exosomes? On primacy, precision, and popularity influencing a choice of nomenclature. J. Extracell. Vesicles 8 (1), 1648167. 10.1080/20013078.2019.1648167 31489144 PMC6711079

[B51] Xiao D. Barry S. Kmetz D. Egger M. Pan J. Rai S. N. (2016). Melanoma cell-derived exosomes promote epithelial-mesenchymal transition in primary melanocytes through paracrine/autocrine signaling in the tumor microenvironment. Cancer Lett. 376 (2), 318–327. 10.1016/j.canlet.2016.03.050 27063098 PMC4869527

[B52] Xie L. Li J. Wang G. Sang W. Xu M. Li W. (2022). Phototheranostic metal-phenolic networks with antiexosomal PD-L1 enhanced ferroptosis for synergistic immunotherapy. J. Am. Chem. Soc. 144 (2), 787–797. 10.1021/jacs.1c09753 34985903

[B53] Zhang D. Qin X. Wu T. Qiao Q. Song Q. Zhang Z. (2019a). Extracellular vesicles based self-grown gold nanopopcorn for combinatorial chemo-photothermal therapy. Biomaterials 197, 220–228. 10.1016/j.biomaterials.2019.01.024 30669014

[B54] Zhang D. Wu T. Qin X. Qiao Q. Shang L. Song Q. (2019b). Intracellularly generated immunological gold nanoparticles for combinatorial photothermal therapy and immunotherapy against tumor. Nano Lett. 19 (9), 6635–6646. 10.1021/acs.nanolett.9b02903 31393134

[B55] Zhang X. Wang C. Wang J. Hu Q. Langworthy B. Ye Y. (2018). PD-1 blockade cellular vesicles for cancer immunotherapy. Adv. Mat. 30 (22), e1707112. 10.1002/adma.201707112 29656492

[B56] Zhang Y. Zhang Z. (2020). The history and advances in cancer immunotherapy: Understanding the characteristics of tumor-infiltrating immune cells and their therapeutic implications. Cell. Mol. Immunol. 17 (8), 807–821. 10.1038/s41423-020-0488-6 32612154 PMC7395159

[B57] Zhou X. Yan T. Huang C. Xu Z. Wang L. Jiang E. (2018). Melanoma cell-secreted exosomal miR-155-5p induce proangiogenic switch of cancer-associated fibroblasts via SOCS1/JAK2/STAT3 signaling pathway. J. Exp. Clin. Cancer Res. 37 (1), 242. 10.1186/s13046-018-0911-3 30285793 PMC6169013

[B58] Zhu L. Gangadaran P. Kalimuthu S. Oh J. M. Baek S. H. Jeong S. Y. (2018). Novel alternatives to extracellular vesicle-based immunotherapy - exosome mimetics derived from natural killer cells. Artif. Cells Nanomed. Biotechnol. 46 (3), S166–S179. 10.1080/21691401.2018.1489824 30092165

[B59] Zhu L. Kalimuthu S. Gangadaran P. Oh J. M. Lee H. W. Baek S. H. (2017). Exosomes derived from natural killer cells exert therapeutic effect in melanoma. Theranostics 7 (10), 2732–2745. 10.7150/thno.18752 28819459 PMC5558565

